# Initial experience using a handheld fully articulating software-driven laparoscopic needle driver in TAPP inguinal hernia repair

**DOI:** 10.1007/s00464-021-08446-6

**Published:** 2021-04-06

**Authors:** Victoria Needham, Diego Camacho, Flavio Malcher

**Affiliations:** 1 Department of Surgery, Montefiore Medical Center, 111 East 210th Street, Bronx, NY 10467 USA; 2 Albert Einstein College of Medicine, Bronx, NY USA

**Keywords:** Inguinal hernia repair, Laparoscopy, Laparoscopic suturing, TAPP, Articulating needle driver

## Abstract

**Background:**

The laparoscopic transabdominal preperitoneal (TAPP) inguinal hernia repair is a widely performed minimally invasive operation, but can present considerable ergonomic challenges for the surgeon. Our objective was to determine if a novel handheld software-driven laparoscopic articulating needle driver can mitigate these difficulties.

**Methods:**

The video recordings of a consecutive series of TAPP cases by a single surgeon using the articulating device were compared with a series of cases using straight-stick laparoscopy. Two critical steps of the procedure were analyzed for time: mesh fixation and peritoneal suture closure. These steps were then compared before and after 10 initial consecutive cases to analyze whether the surgeon demonstrated improvement. A cost analysis was also performed between the two techniques.

**Results:**

For mesh fixation, the surgeon averaged 227 s using tacker devices, compared with 462.4 s using the novel laparoscopic device (*p* = 0.06). For the peritoneal closure component of the operation, the surgeon improved the time per suture pass during closure from 60.61 s during the first 10 cases to 38.84 s after the first 10 cases (*p* = 0.0004), which was comparable to the time per stitch for standard laparoscopy (34.8 s vs 34.84 s, *p* = 0.997). Left-sided inguinal hernia repairs using the articulating device demonstrated a significantly longer time per stitch during peritoneal closure compared to the right side after first 10 cases (left: 40.62 s; right: 27.91, *p* = 0.005). Our direct cost analysis demonstrated that suture closure of the peritoneum using the articulating device was more cost-effective than tack fixation.

**Conclusions:**

After only a 10 case initial experience, a laparoscopic hand-held articulating needle driver is comparable to standard laparoscopy to complete suture mesh fixation and peritoneal closure for TAPP inguinal hernia repair. Further, the feasibility of suture mesh fixation minimizes the need for costly tacker devices. This instrument appears to be a promising tool in this largely minimally invasive era of hernia repair.

There is an emerging market for advanced laparoscopic devices that bridge the gap between traditional straight-stick laparoscopic equipment and robotic platforms. Specifically, advanced hand-held grasping forceps and needle drivers have emerged. These devices cite several advantages, from simple mechanical enhancements that provide wrist-like range of motion, to imbedded software that aims to improve both ergonomics and surgical precision [1]. In this study, we analyze the performance of the HandX™, a novel handheld software-driven laparoscopic needle driver with an articulating head, during the TAPP inguinal hernia repair.

The HandX™ device from Human Xtensions is an FDA-approved hand-held advanced laparoscopic modular platform with an interchangeable articulating head that fits through 5 mm ports (Fig. 1). Its computerization allows the surgeon to translate basic hand movements into complex directions and motions inside the surgical field. The device is quick to set up and requires minimal staff training. Other potential advantages include the same ergonomic benefits touted by other advanced hand-held devices, as it is light-weight and can be used entirely with one hand, and allows for potentially greater freedom of port placement compared to traditional straight-stick laparoscopic triangulation. Additionally its integrated wireless technology is able to collect and transmit data regarding its use and maintenance [2].

**Fig. 1:**
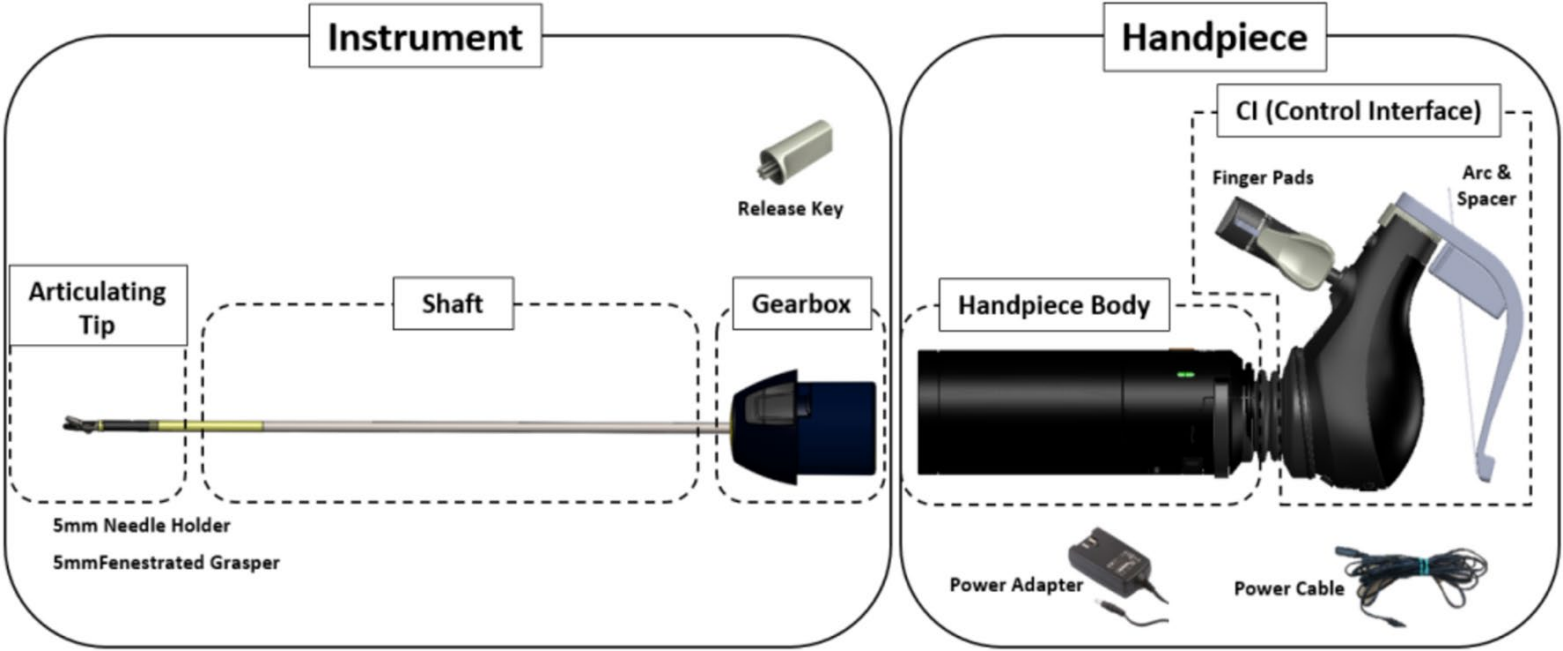
HandX™ device by Human Xtensions

The TAPP technique for inguinal hernia repair is a widely used minimally invasive approach for repairing inguinal hernias. This operation was chosen to analyze the performance and feasibility of the HandX™ device for several reasons. Inguinal hernia repair is a common operation with over 20 million procedures performed annually [3]. 25% or greater of all inguinal hernias are repaired laparoscopically, and there is clear evidence in the literature for the benefits of faster return to work and lesser postoperative and chronic groin pain with the minimally invasive approach compared to an open approach [4]. The two laparoscopic techniques for inguinal hernia repair are TAPP or total extraperitoneal repair (TEP). TAPP touts the advantages of looking at and dissecting all potential groin hernia spaces and anatomy from a further distance and direct visualization of the hernia. The choice between the two techniques is largely based on surgeon training and preference [4]. The TAPP is performed by establishing pneumoperitoneum, creating a preperitoneal plane, dissecting the potential femoral and inguinal hernia sacs out and free from other structures, and once reduced, placing a mesh in the preperitoneal space and closing the peritoneal flap to entirely separate the mesh from the intraabdominal contents [5].

Frequently the mesh is fixated to the anterior abdominal wall prior to closure of the peritoneum [3]. With straight-stick laparoscopy this is frequently performed using a laparoscopic tacker device. Working in the narrower lower abdomen with standard laparoscopy makes both the suture fixation of the mesh and closure of the peritoneal flap both ergonomically challenging and time-consuming. Therefore the TAPP provides an adequate platform to compare straight-stick laparoscopy and the HandX™ device head-to-head for these two key components of the TAPP procedure.

The goals of this study were to elucidate the feasibility of HandX™ for both fixation of the mesh as well as peritoneal closure during TAPP repair, and to compare the handling and timing of the device with straight-stick laparoscopy for TAPP repair. We also aimed to perform a basic direct cost-analysis between the two methods for this specific surgery. We hypothesize that the HandX™ and similar advanced laparoscopic devices are starting to create a niche market between the 40 year old traditional laparoscopic equipment and the much more labor- and cost-intensive available robotic platforms.

## Materials and methods

**Fig. 2:**
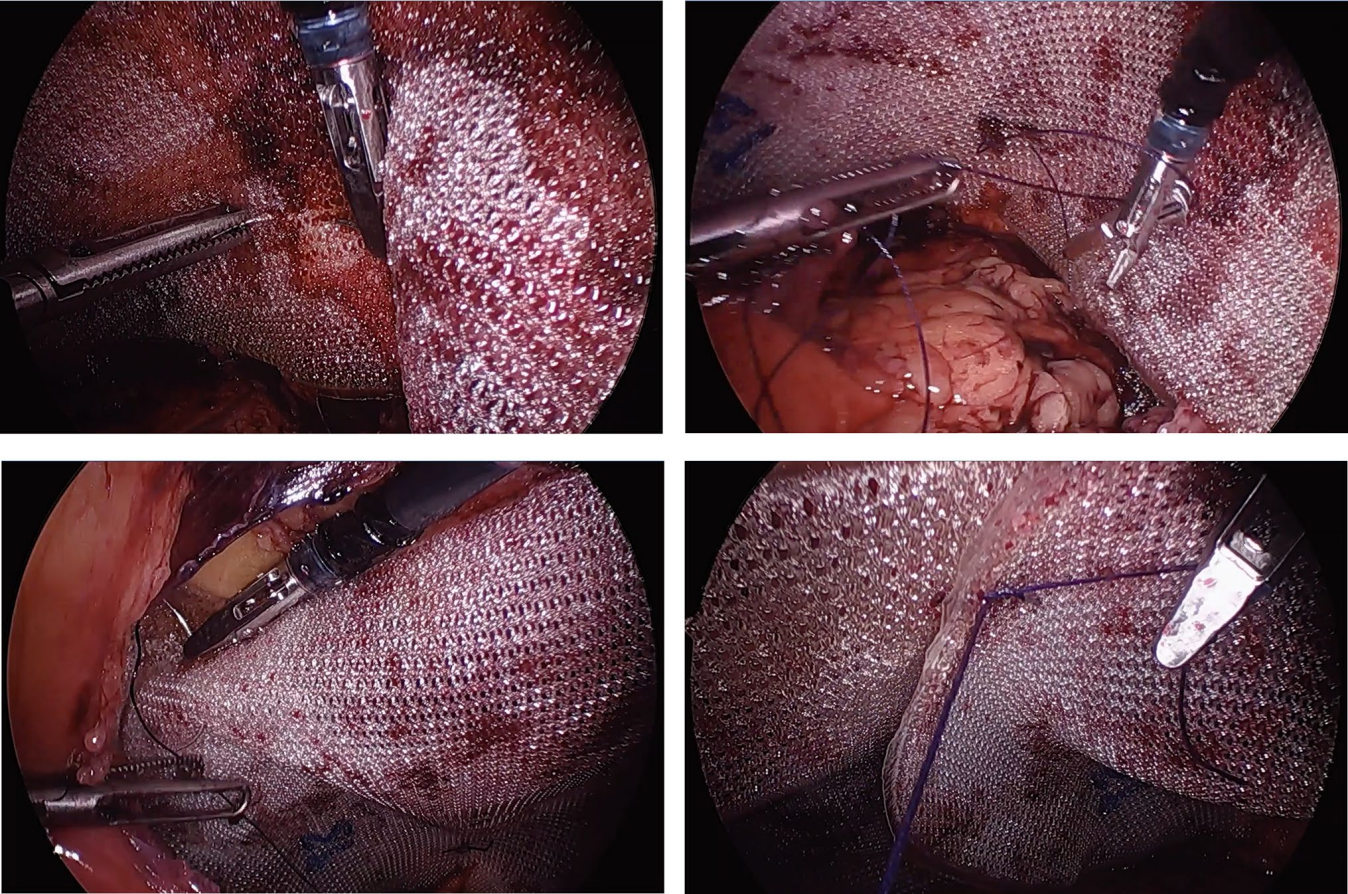
Suture mesh fixation during TAPP repair using the HandX™ device

**Fig. 3:**
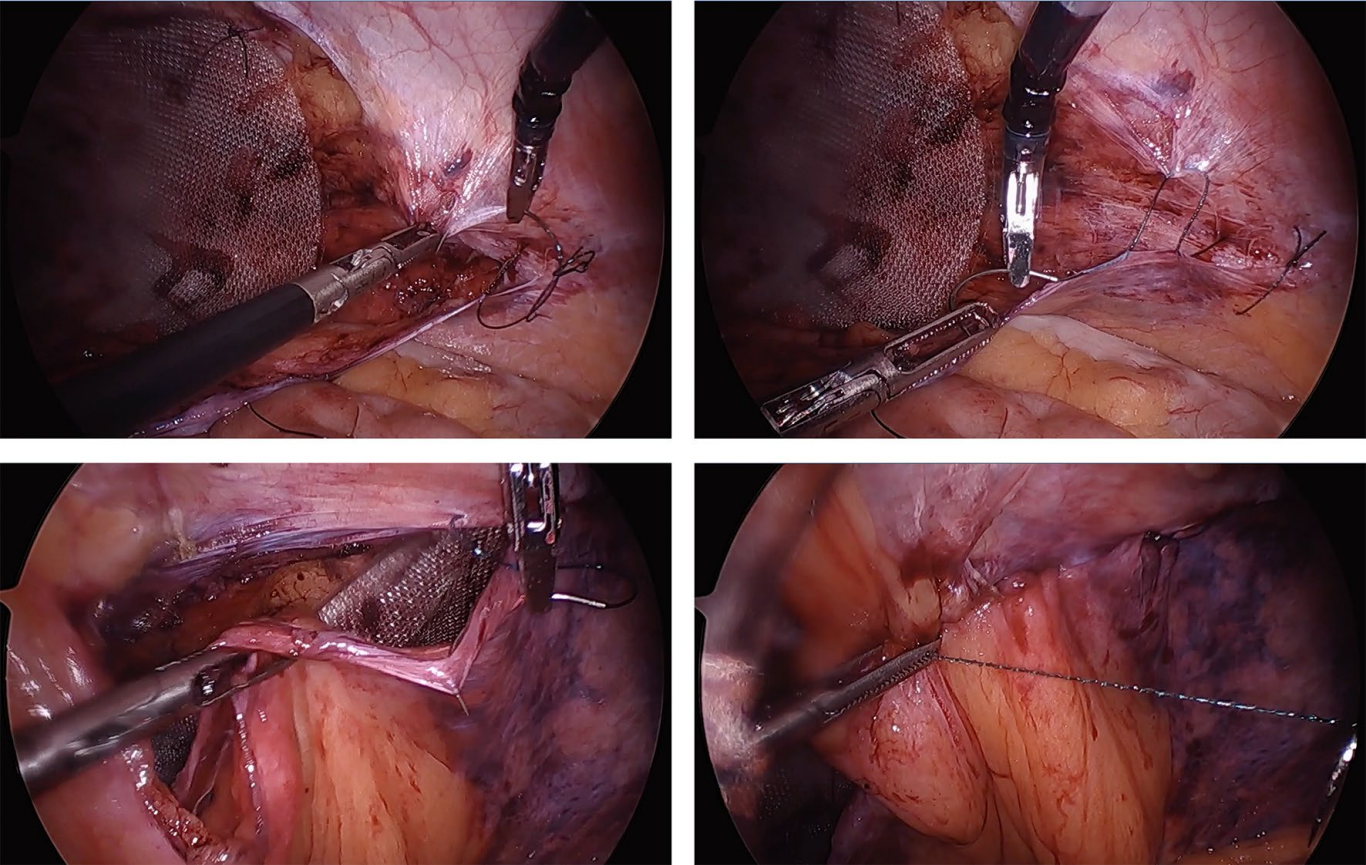
Peritoneal closure during TAPP repair with horizontal mattress suture using HandX™ device

**Fig. 4:**
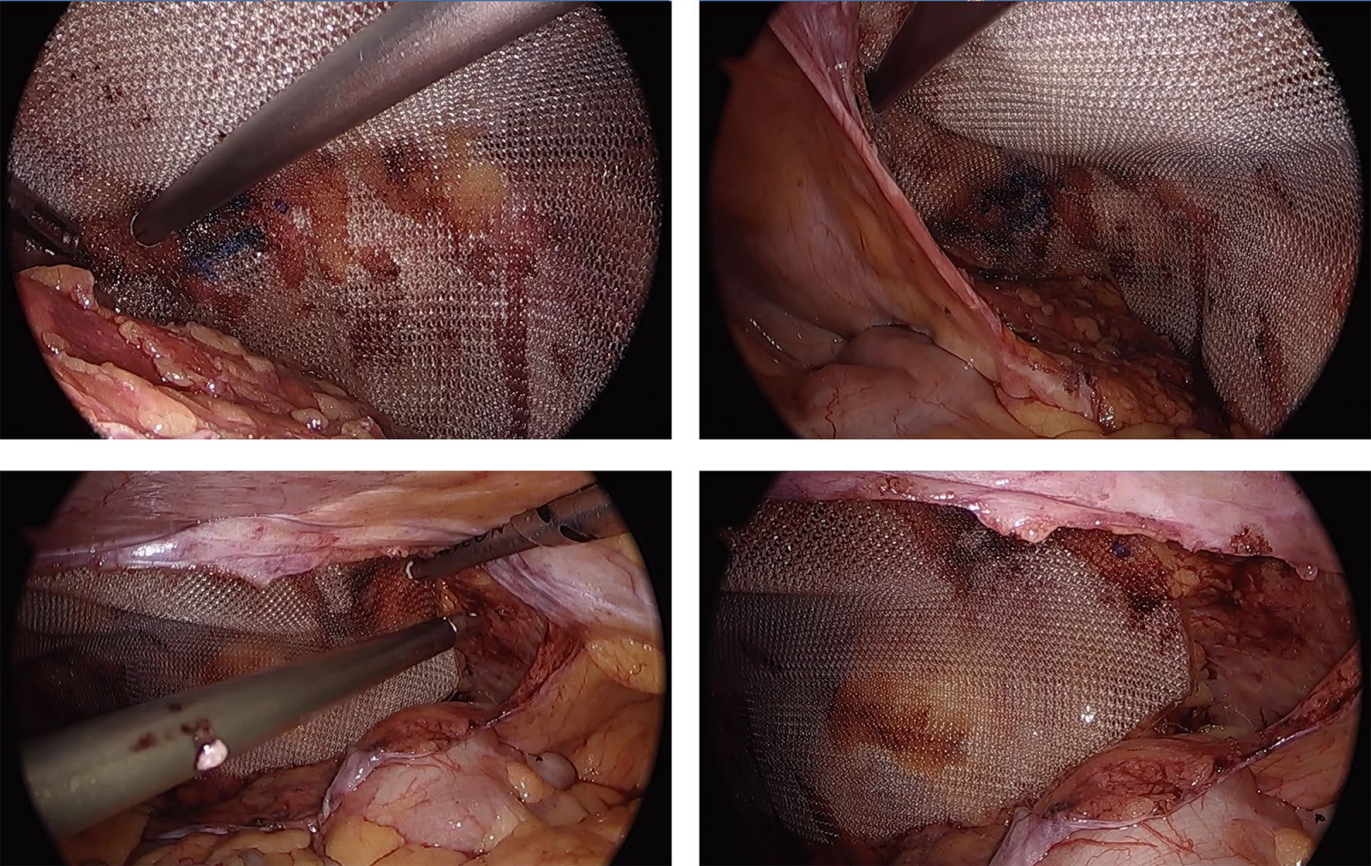
Mesh fixation during TAPP repair using laparoscopic tacking device

**Fig. 5:**
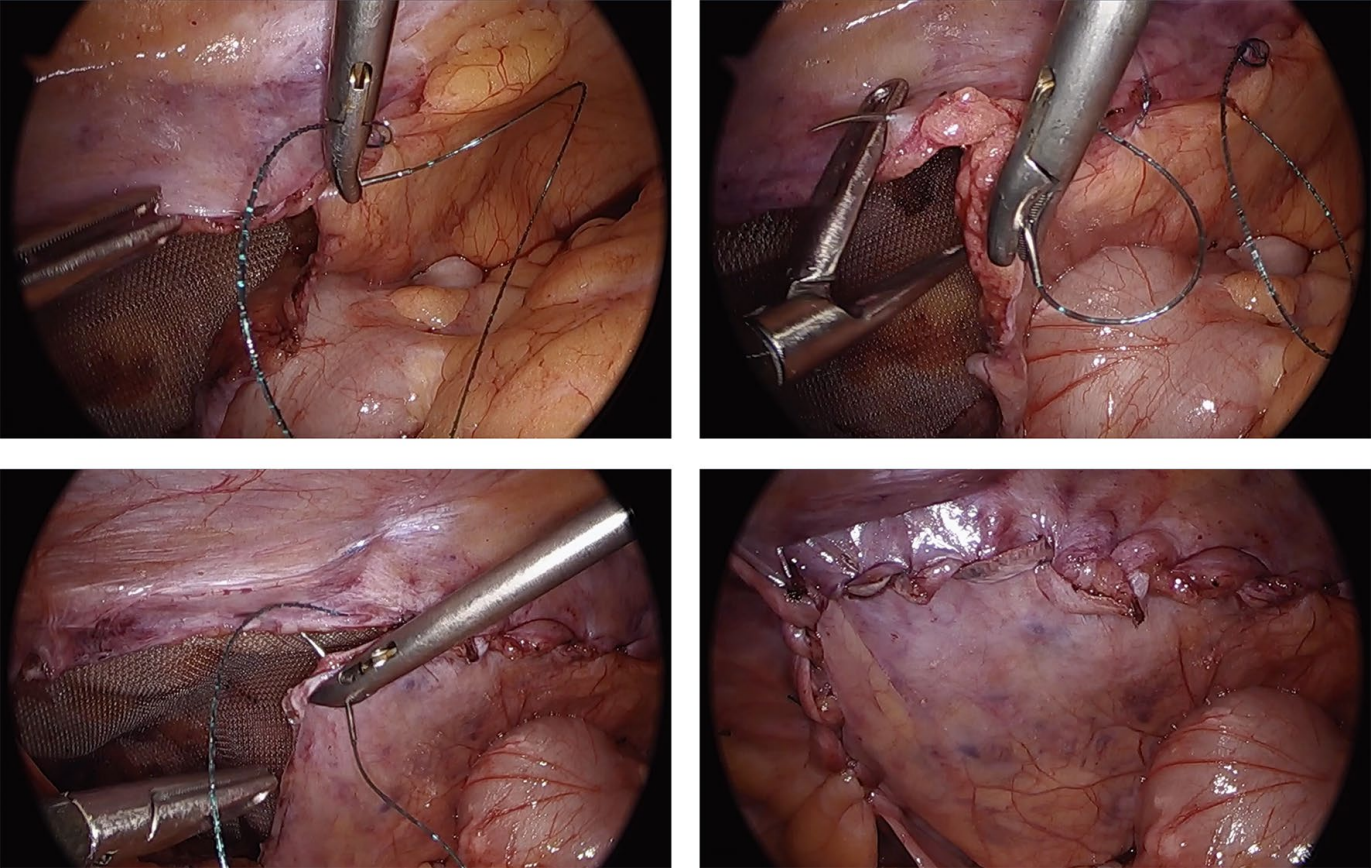
Peritoneal closure during TAPP repair with running baseball suture using standard laparoscopic technique

This study was IRB-approved and HIPAA compliant. A single right hand dominant surgeon performed a consecutive series of TAPP inguinal hernia repairs employing the HandX™ device, which were video-recorded. In these cases, the HandX™ laparoscopic needle-holder was used for the entirety of suturing three interrupted sutures (3-0 Vicryl suture, ETHICON™) of the mesh to the abdominal wall (two medial and one lateral, as shown in Fig. 2), and for a running horizontal mattress closure of the peritoneal flap using a barbed/locking suture (2-0 VLock suture, Medtronic) (Fig. 3). The videos using the HandX™ were then compared with a recent consecutive series of traditional straight-stick laparoscopic TAPP inguinal hernia repairs performed by the same surgeon. In the straight-stick laparoscopic TAPP repairs the mesh was fixated using a disposable tacker device (Fig. 4), and the peritoneal flap was closed using a running baseball-style stitch (2-0 VLock suture, Medtronic) (Fig. 5).

The videos from both techniques were then evaluated by an independent reviewer to collect the following quantitative variables: time of mesh fixation (in seconds), time of closure of the peritoneum (in seconds), number of suture passes through the peritoneum required to close the flap, and time per suture pass (in seconds). The analysis was then stratified to compare the first 10 consecutive HandX™ cases with the subsequent consecutive case series. The data were also separated into right- and left-sided hernia repairs. In cases of simultaneous bilateral inguinal hernia repair, the right and left sides were considered as different cases, and the surgical steps were analyzed independently.

The primary endpoint was time per stitch to fixate the mesh and to close the peritoneal flap. Secondary endpoints were total time of either task, total number of stitches using either method, right- vs left-sided repairs, and components of closure of the peritoneal flap before and after the first 10 cases. Where noted, the “number of stitches” for peritoneal flap closure refers to each complete suture pass of the needle through both sides of the peritoneum for the running or horizontal mattress style suture closure. “Time per stitch” refers to the time from the physical entry of the needle into the peritoneum to begin one suture throw, all the way up to just before the entry of the subsequent pass through the peritoneum. Quantitative variables were compared using unpaired *t*-tests using Microsoft Excel Data Analysis package.

Additionally a basic direct cost analysis was performed between a standard laparoscopic approach to TAPP repair (using disposable tacks to fixate the mesh and suture to close the peritoneum), and the TAPP repair using the HandX™ device (fixation and closure accomplished with sutures). Suture material was not included in the cost analysis as it overall incurred nominal cost compared to the devices employed. Cost per unit of tacker devices was obtained from the medical record as billed during the surgical case. Cost for HandX™ device was obtained from company literature, as a calculation of cost per operation taking into account the service cost and cost of the disposable part employed specifically for TAPP repair, the needle driver tip (see Table 5). The capital cost was calculated by the company over two different usage points, 200 or 300 cases per year per institution.

## Results

During the study period, the surgeon analyzed the step of mesh fixation in 21 cases, 3 with standard laparoscopic technique and 18 using the HandX™ device, and the surgeon analyzed peritoneal closure in 27 total repairs, 8 with standard laparoscopy and 19 using HandX™. The surgeon on average required 227.00 s to fixate the mesh using a laparoscopic tacker device, compared with 462.39 s to suture fixate the mesh using the articulating handheld laparoscopic needle driver (*p* = 0.059). For peritoneal closure, the time to perform flap closure was comparable between the two techniques (594.63 s for pure laparoscopic running baseball closure vs 720.26 s for horizontal mattress closure using the HandX™ device, *p* = 0.084). The surgeon employed a significantly fewer number of sutures to close the peritoneum during the first 10 cases using the HandX™ device (17.25 using straight-stick laparoscopy vs 13.00 with HandX™, *p* = 0.047), and took a significantly longer time per stitch during these first 10 cases (34.83 s vs 60.61 s, *p* = 0.0005). Taken altogether, there was a significantly faster time per stitch observed for straight-stick laparoscopy closure of the peritoneum versus use of the HandX™ device (34.8 s vs 45.69 s, *p* = 0.016). However, after the initial 10 cases, no difference was appreciated between the two groups for time per stitch of peritoneal flap closure (34.8 vs 34.84, *p* = 0.997). When this data were stratified by comparing the first 10 cases of peritoneal closure using HandX™ to the subsequent cases, again we appreciated that the surgeon employed a significantly greater number of stitches after 10 cases (13.00 during the first 10 cases vs 19.45 after 10 cases, *p* = 0.0002), but actually significantly improved the time per stitch using the HandX™ in the later cases (60.61 s during the first 10 cases vs 38.84 s after the first 10 cases, *p* = 0.0004). When peritoneal suturing using the HandX™ device was stratified by side, the time per stitch remained significantly improved after the initial 10 cases only for right-sided suturing. This data are summarized in Tables 1 and 2.

**Table 1:** Comparing standard laparoscopic technique for TAPP hernia repair (tack fixation and suture closure of the peritoneum) vs assisted by the HandX™ device (suture fixation of the mesh and suture closure of the peritoneum) across a variety of metrics: mesh fixation time, peritoneal closure time, number of stitches (suture passes) during peritoneal closure, and time per stitch of peritoneal closure

	Standard laparoscopy	HandX device	*p*
Mesh fixation			
Mesh fixation (*t*)	227	462.39	0.059
Mesh fixation after initial 10 HandX™ cases (*t*)	227	403.27	0.106
Peritoneal closure			
Time of closure all cases (*t*)	594.63	720.26	0.084
Time of closure during first 10 HandX™ cases (*t*)	594.63	779.88	0.063
Time of closure after first 10 HandX™ cases (*t*)	594.63	676.91	0.281
Number of stitches all cases	17.25	16.74	0.788
Number of stitches during first 10 HandX™ cases	17.25	13.00	0.047
Number of stitches after 10 HandX™ cases	17.25	19.45	0.230
Time per stitch all cases (*t*)	34.83	45.69	0.016
Time per stitch during first 10 HandX™ cases (*t*)	34.83	60.61	0.0005
Time per stitch after first 10 HandX™ cases (*t*)	34.83	34.84	0.997

**Table 2:** Metrics of suture closure of peritoneum using the HandX™ device, comparing the first 10 cases with subsequent cases

	First 10 HandX™ cases	After 10 HandX™ cases	*p*
Peritoneal closure			
Time of closure (*t*)	779.88	676.91	0.273
Number of stitches	13.00	19.45	0.0002
Time per stitch (*t*)	60.61	38.84	0.0004
Right sided			
Time of closure (*t*)	676.00	535.60	0.151
Number of stitches	12.00	19.20	0.005
Time per stitch (*t*)	57.13	27.91	0.004
Left sided			
Time of closure (*t*)	953.00	794.67	0.174
Number of stitches	14.67	19.67	0.030
Time per stitch (*t*)	66.40	40.62	0.117

When comparing performance of standard laparoscopic suturing for TAPP repair between right and left sides, there was no significant difference in time of peritoneal closure, number of stitches, and time per stitch between sides. However, when using the HandX™ device, left-sided repairs took significantly more time than right-sided repairs (847.44 s vs 605.60 s, *p* = 0.002). When stratified by first 10 cases vs subsequent HandX™ cases, this difference was significant only for the later cases. Similarly the time per stitch for peritoneal closure using HandX™ was significantly longer for the left side than the right side after the initial 10 cases (40.62 s vs 27.91 s, *p* = 0.005). These data are summarized in Tables 3 and 4. Mesh fixation was not stratified by right- and left-sided repairs.

**Table 3:** Metrics for peritoneal closure across both techniques stratified by right and left anatomical hernia repair

Peritoneal closure	Right	Left	*p*
Standard laparoscopy (*t*)	578.50	610.75	0.791
Standard laparoscopy number of stitches	16.75	17.75	0.793
Standard laparoscopy time per stitch (*t*)	34.81	34.85	0.991
HandX™ all cases (*t*)	605.80	847.44	0.002
Number of stitches all cases	15.60	18.00	0.192
Time per stitch all cases (*t*)	42.52	49.21	0.392
First 10 HandX™ cases (*t*)	676.00	953.00	0.055
After 10 HandX™ cases (*t*)	535.60	794.57	0.004
Number of stitches first 10 HandX™ cases (*t*)	12.00	14.67	0.195
Number of stitches after 10 HandX™ cases (*t*)	19.20	19.67	0.679
Time per stitch first 10 HandX™ cases (*t*)	57.13	66.40	0.441
Time per stitch after 10 HandX™ cases (*t*)	27.91	40.62	0.005

**Table 4:** Metrics for peritoneal closure using standard laparoscopy vs the HandX™ device for either anatomical right- or left-sided hernia repair

	Standard laparoscopy	HandX device	*p*
Right side			
Time of closure (*t*)	578.50	605.80	0.669
Number of stitches	16.75	15.60	0.451
Time per stitch	34.81	45.52	0.251
Left side			
Time of closure (*t*)	610.75	847.44	0.113
Number of stitches	17.75	18.00	0.948
Time per stitch	34.85	49.21	0.030

In our cost analysis, we compared the cost per unit (as noted in our hospital’s electronic medical record) for tack fixation with standard laparoscopic technique vs suture fixation using the HandX™ device (Table 5). Mesh type was the same across both techniques, only varying slightly between choice of “light” vs regular lightweight polypropylene mesh of the same brand, and choice of large or extra-large and right- or left-sided version depending on patient anatomy. The cost of the HandX™ device per operation was determined by factoring in the capital, service and instrument expenses across either 200 or 300 annual procedures. Altogether, the HandX™ technique demonstrated a cost savings coming in at a range of $520–$580 per procedure compared to standard laparoscopy ranging from $620.94–$730 per case.

**Table 5:** Cost analysis of major devices employed during TAPP hernia repair with either standard laparoscopic technique (employing tack fixation of the mesh) or HandX™-assisted technique (employing suture fixation of the mesh)

TAPP, standard laparoscopic technique—tacker fixation of mesh	
	Average cost per operation (USD)
Disposable laparoscopic tacker^a^	$488.27

TAPP, Handx™-assisted laparoscopic technique—suture fixation of mesh				
# Annual procedures	Capital expense^b^	Service expense^b^	Instrument expense^b^	Total cost per operation (USD)^b^
200	50.00	10.00	310.00	$370.00
300	33.33	6.67	310.00	$350.00

Lightweight polypropylene mesh (suture or tack fixation)	
Mesh	Average cost per operation (USD)
Laparoscopic hernia mesh^c^	$190.53

Combined cost	
Fixation and peritoneal closure technique	Price range
Laparoscopic hernia mesh + tacking device (standard laparoscopic technique)	$620.94–$730.00
Laparoscopic hernia mesh + suture fixation (HandX™ assisted technique)	$520–$580

^a^Cost variation of maximum $50 per procedure at our institution regardless of brand

^b^Cost of HandX™ per number of annual procedures

^c^Cost variation of maximum $40 per procedure at our institution regardless of brand/size

## Discussion

The Human Xtensions HandX™ is an electromechanical handheld modular laparoscopic device with an articulating elbow. The Control Interface contains easily accessible buttons that allow the surgeon to translate combinations of one-handed motions and commands to complex actions within the laparoscopic surgical field along the Handpiece Body and into the disposable and interchangeable articulating effector tip. In our study, we focused on the use of the needle driver tip attachment. Specifically, we demonstrated that the HandX™ fitted with the needle driver tip allows the surgeon to access complex angles to drive the needle along the anterior abdominal wall to both tack the mesh within the preperitoneal space and to close the peritoneum in TAPP inguinal hernia repair. We found that the device offers a cost-effective alternative to tacker fixation of the mesh and is comparable in timing to straight-stick laparoscopy for peritoneal closure, measured in time per stitch, after a 10 case initial experience. Both techniques were compared over two metrics: total time to closure as well as time per stitch. These were compared for both the first 10 cases and after the first 10 cases with the HandX™. The time per stitch metric was chosen in addition to the time per closure because we found several limitations with total time of closure. First, every patient varies in the size of peritoneal flap to close, translating to different length of time needed to close the flap. Also, along with a shorter total time to closure as the surgeon learns to use the HandX™, we also observed that the surgeon trended toward a larger number of stitches to close the peritoneum as time went on, which may reflect additional mastery of the device; we hypothesize as the surgeon gains more fine motor skills with the device, they are able to place finer and more accurate sutures. Therefore, time per stitch allows standardization of the surgeon’s performance across the entire series.

The intraperitoneal motions employed in the TAPP repair were made possible by the HandX™ with less wrist and arm motions on the surgeon’s part outside of the body compared to straight-stick laparoscopy, as the effector tip provided the articulation via the control interface, and not the surgeon directly. As noted by Szold et al., straight-stick laparoscopic ports create a fixed fulcrum at the trocar entry site which allow only four degrees of freedom (DoF) within the cavity, as opposed to their natural six DoF outside of the body. When an articulating effector is added to the instrument, as is common with the advanced handheld laparoscopic devices on the market, the surgeon regains these two DoF that exist with a normal wrist motion in open surgery [1]. This mechanical advantage translates into ergonomic advantages for the surgeon when performing a TAPP repair, in the ability to suture more easily at the anterior abdominal wall, and to cross the midline from existing ports. In addition to angulation the HandX™ also provides up to 270° of rightward and 90° of leftward rotation, independent of surgeon wrist rotation as well as a locking mechanism to work from any given degree of rotation, which was employed in our study during peritoneal closure, allowing for even greater range of motion [6].

During TAPP repair, the mesh is commonly anchored within the preperitoneal space to the underlying fascia to increase its stability during abdominal desufflation and thereby reduce the recurrence rate. Specifically the fixation of the mesh at its medial aspect at Cooper’s ligament has been shown to be most significant in terms of reducing recurrence. Fixation can be accomplished with tackers, staples, sutures or fibrin glue [7]. Alternatively a self-gripping mesh can be utilized with or without additional fixation at Cooper’s ligament [3]. Both absorbable and nonabsorbable tacks have been documented in the literature to incur greater postoperative pain [7]. Specifically both the genital and femoral branches of the genitofemoral nerve and the lateral femoral cutaneous nerve have been implicated especially when fixation is performed in the lateral surgical field [8]. Additionally Lantis and Schwaitzberg published a case report of a screw-type tacker directly penetrating the ilioinguinal nerve requiring reoperation and extraction [9]. While the available randomized studies regarding mesh fixation are heterogenous, the current guidelines from both the International and European Hernia Societies advise to use the least traumatic method possible and necessary for the given hernia repair [3, 10]. In our experience, the articulating tip of the HandX™ device allowed for suture fixation of the mesh against the anterior abdominal wall deep within the preperitoneal space. As this maneuver is ergonomically challenging with straight-stick laparoscopy, we utilize tacks for fixation in those cases. Not only does this introduce the concern of greater postoperative pain, it also incurs a greater cost, as demonstrated in our cost-analysis table.

**Fig. 6:**
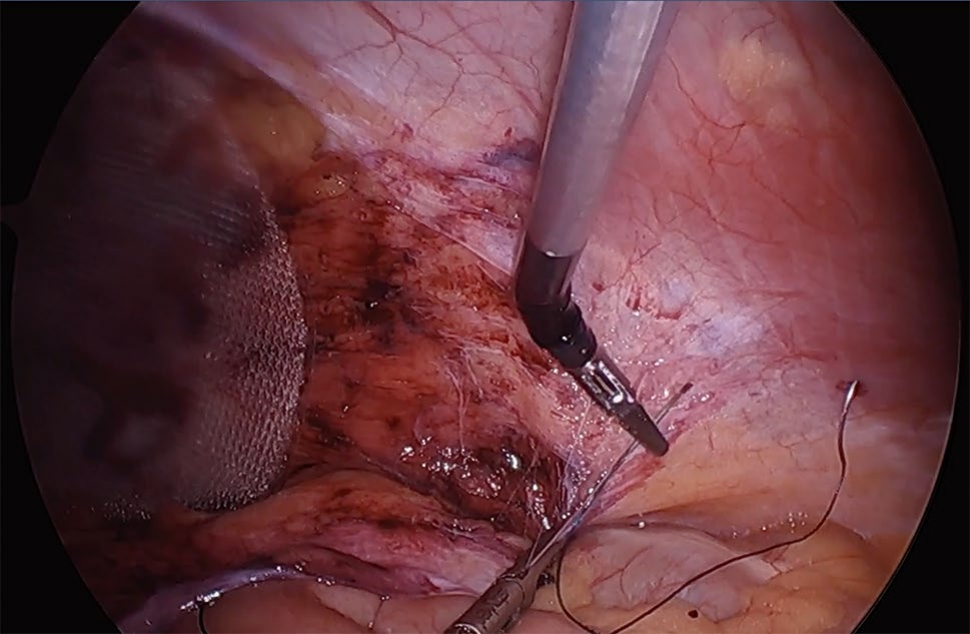
Utilization of the articulating elbow of the HandX™ device to facilitate laparoscopic suturing at the anterior pelvic wall

We traditionally perform the peritoneal closure during the TAPP repair with a running baseball-type suture [5]. This step involves significant ergonomic challenges given the limited DoF with straight-stick laparoscopic instruments. As illustrated in our study, even an experienced minimally invasive surgeon may take over 30 s on average to complete one peritoneal stitch. Surgeons may circumvent this challenge by employing tacks to close the peritoneum as an alternative. However, this is not without risk of acute and chronic pain, similar to the tack fixation of the mesh. Heniford et al. demonstrated that over 10 tacks in the anterior abdominal wall can incur reduced quality of life compared to nonfixated or sutured meshes in TEP and open inguinal hernia repair [11]. In a more recent randomized study comparing tacks to fibrin glue and suture fixation, Heniford et al. further demonstrated significant reduction in QOL in the tacker group [12]. Barbed suture material such as the VLoc provides a small ergonomic benefit in the way of eliminating the need for suture tying and maintaining tension between bites without needing additional retraction; however this suture material is also not without risk, as numerous case reports describe the incidence of small bowel obstruction directly caused by exposed barbed suture material [13, 14]. In our experience, not only does the HandX™ allow for greater DoF to perform the suture closure, it also allows the surgeon to perform a more complex horizontal mattress closure which in turn ensures that all barbed suture material is buried. As seen in Fig. 6, with the elbow of the HandX™ articulating down towards the pelvis, there is very little other motion needed to load the needle or to pull the suture through between each stitch in this complex horizontal mattress suture. We also observed a significantly larger number of suture passes necessary to close the peritoneum with the HandX™ after the initial 10 case experience. We hypothesize that this may be secondary to the increased ease of suturing, and may lend itself to a more adequate closure, via smaller gaps between sutures. As the angulation of the pelvis creates the greatest peritoneal flap tension for the TAPP repair to occur at the midline, the flap is generally and in our case always sewn from lateral to medial. This translates to a standard forehand stitch for a right-handed surgeon on right-sided TAPP repairs, and a “back hand” approach for the right-handed surgeon in left-sided TAPP repairs. This difference along with surgeon’s ongoing learning curve on the HandX™ device may explain the significant difference in time per stitch between right- and left-sided repairs with the device as compared to standard laparoscopic peritoneal closure. As we note in Table 3 however, although the left remains slower than the right, all time metrics are improved for both hands after the first 10 cases with HandX™, and so additional data over a longer series of cases would be required to determine if the device has any true limitations on the surgeon’s non-dominant side.

We demonstrated that the greater range of motion provided by the HandX™ device translates to greater DoF in the intraperitoneal cavity. This allowed for comparable operative times. In turn, we hypothesize that the greater DoF translates to improved ergonomics for the operating surgeon. Multiple studies have demonstrated this outcome with other handheld advanced laparoscopic devices, using ergonomic gloves and electromyographic sensors to measure the workload on various muscles (the RULA method or Rapid Upper Limb Assessment) [15]. The literature and workplaces have cast more attention on the comfort and safety of the operating surgeon in this rapidly expanding minimally invasive era. While our study demonstrates the surgeon’s ability to manipulate the HandX™ for use in TAPP repair, more dedicated ergonomic studies would be useful to collect qualitative and quantitative data regarding the specific motions of both the surgeon and the HandX™ in this operation as compared to straight-stick laparoscopy, and also in comparing the surgeon’s experience with right versus left-sided repairs.

Our cost analysis demonstrated the cost-effectiveness of employing the HandX™ device in a TAPP repair for suture fixation of the mesh compared to standard laparoscopic technique employing tacker fixation. The HandX™ device is also unique in its modular interchangeable tip, which allows for only one disposable component of the device. Additionally the platform itself does not need to be sterilized with each use; rather it is placed into a sterile disposable sleeve for each case. With a capital cost the fraction of a robotic platform, after only 4–5 cases per week (200–300 cases per year), realistic for a busy hernia center, the HandX™ is a viable way to provide additional operative versatility and avoid costly tacker devices. As with other minimally invasive platforms including robots, the cost benefits take several years to manifest after the capital costs are paid off. More rigorous cost analysis over an institution or health system instead of a single surgeon, taking into account length of surgery, length of hospital stay, and any postoperative complications may be more comprehensive if the adaptation of advanced laparoscopic platforms becomes more widespread.

Certainly a robotic platform can provide complex visualization and articulation along with favorable ergonomics for a range of intraperitoneal procedures. However, with the high capital cost of a robotic platform along with the extensive surgeon and staff training required, the market for an advanced handheld device is imperative and growing. Future studies should focus specifically on the performance, cost and ergonomics of the HandX™ device compared to a robotic platform for the TAPP repair as well as other commonly performed minimally invasive procedures. As stated by Szold et al., the advanced handheld devices provide tactile feedback which may translate to more precision operating as compared to a robot [1]. Therefore, future studies investigating precision and accuracy regarding specific surgical techniques may also be warranted.

### Limitations

The data provided reflects a small sample size for a single surgeon. It is retrospective and analyzes two specific portions of one operation. It should be noted that the HandX™ and its comparable devices on the market do involve a considerable training period and are not simply intuitive for a well-trained surgeon with a minimally invasive skill set. This is reflected by the time/stitch data of over 60 s in our early cases. A larger series over a longer learning curve with more surgeons of different experience levels would be useful in gaining stronger data. Additionally the learning curve was performed over consecutive cases over several months, and may be affected by repetition in such a short time. The surgeon is right-handed, which may have influenced the right versus left-sided hernia repair analysis. Time to closure, time/stitch, and a cost-analysis provided concrete quantitative metrics to compare the HandX to standard laparoscopy; however the decision for a surgeon or hospital to invest in the HandX™ platform would likely be based on a combination of this data as well as additional qualitative ergonomic data as well as patient outcomes.

Working in the narrower lower abdomen and pelvis with standard laparoscopic equipment presents well-documented ergonomic challenges, which has led pelvic specialties like OB-GYN and Urology to rely heavily on robotic platforms for their increased DoF and angulated instruments. Similarly peritoneal flap closure in a TAPP repair, a critical step of the procedure, presents significant challenges. Laparoscopic inguinal hernia repair is a frequent procedure for most general and minimally invasive surgeons. Therefore we felt this operation to be the most useful arena to test the HandX™ device. In future studies the device should be employed to compare fascial closure or mesh fixation in other operations such as intraperitoneal onlay mesh ventral hernia repairs, as these also require suturing at the anterior abdominal wall, and other complex laparoscopic procedures.

This study suggests the concept of establishing a learning curve for advanced laparoscopic devices, as previously described [1]. In other studies of advanced handheld devices, experienced surgeons were observed to either underutilize the rotational aspects of a device via rotation of their own wrist, or overutilize unnecessary aspects of the device to carry out the task at hand. Once these operative steps were streamlined with experience, the learning curve was overcome and the device was found to be as or more efficient than standard laparoscopic technique [15]. After 10 cases, the HandX™ device is comparable to standard laparoscopy to compete suture mesh fixation and peritoneal closure for TAPP inguinal hernia repair. This measure of 10 consecutive cases is not a fixed learning curve nor a plateau; rather it simply illustrates in a single series by one surgeon the potential the device has to become a useful tool not only for its ergonomic benefits in a challenging procedure such as the TAPP but also for the surgeon to not lose valuable time in the operating room. There are more statistically sophisticated methods to demonstrate acquisition of proficiency, such as the longitudinal data analysis technique described in comparing standard laparoscopy to SILS by Sodergren et al. which demonstrated the correlation of the response of specific dependent variables relating to skills acquisition over repeated sessions [16], as well as the linear regression model used by Ramaswamy et al. to describe the change in operative times over the course of 1 year across several surgeons using robotic platform for umbilical hernia repair [17]. This type of study could be conducted in a much larger series of cases among several surgeons, which was not feasible in our case. Additionally, as the device is new to market, it was observational to determine timing of specific steps for one surgeon, verified by one observer and one investigator. This is a useful start to investigate the practical aspects of the device, but again a larger series among several surgeons would help reduce biases.

The HandX™ and other similar advanced laparoscopic devices require significant surgeon and institution buy-in up front and initial training period for staff which can incur additional cost. No untoward outcomes were observed in any of the cases, however, and the safety of this and similar devices is documented across the literature.
